# Preoperative Chemoradiation (Modified Eilber Protocol) Versus Preoperative/Postoperative Radiotherapy for Soft Tissue Sarcomas: A Population-Based Analysis

**DOI:** 10.3390/curroncol32070374

**Published:** 2025-06-26

**Authors:** Greg M. Padmore, Elizabeth C. Kurien, Michael J. Monument, Lloyd Mack, Antoine Bouchard-Fortier, on behalf of the ISARP Group

**Affiliations:** Division of Surgical Oncology, Department of Oncology, Cumming School of Medicine, University of Calgary, Calgary, AB T2N1N4, Canada

**Keywords:** soft tissue sarcoma, radiation, chemoradiation, Eilber protocol

## Abstract

Background: Local recurrence for high-risk extremities/trunk soft tissue sarcoma (STS) after treatment can range from 15 to 30%. The modified Eilber protocol (MEP) using low-dose intravenous chemotherapy with a reduced dosage of radiation in the preoperative setting has demonstrated excellent local control and reduced wound complications in these patients. The aim of the current study was to assess long-term local control and overall survival in patients with STS treated with the MEP versus standard preoperative or postoperative radiotherapy. Methods: Patients diagnosed with STS from 2004 to 2016 were identified using the Alberta Cancer Registry. Patients with STS treated with the MEP, preoperative or postoperative radiotherapy, were included. Patient and tumor characteristics, treatments and outcomes were abstracted from the registry and primary chart review. Characteristics were compared using one-way ANOVA for continuous variable and chi-square test and Fisher test for the categorical outcomes. Local recurrence-free survival and overall survival were analyzed using Kaplan–Meier Analysis with Log-rank test. Results: A total of 242 patients with STS were included, among which 100 (41.3%) received the MEP prior to surgery, 91 (37.6%) had preoperative radiation, and 51 (21.1%) had postoperative radiation. After a median follow up of 4.9 years, there were no significant differences in local recurrence or local recurrence-free survival between patients treated with the MEP vs. preoperative or postoperative radiotherapy (10 vs. 6.6% and 7.8%, respectively, *p*-value NS). There were also no significant differences between groups for recurrence-free survival and overall survival. Conclusions: This study demonstrates that the use of the MEP has non-inferior oncologic outcomes compared to standard preoperative or postoperative radiation in a population-based analysis despite reducing the overall dosage of radiation administered. The modified Eilber preoperative chemoradiation protocol may be considered as an additional option for patients with STS.

## Simple Summary

Soft tissue sarcomas are rare cancers that can occur in the extremities or trunk and are usually treated with limb sparing surgery. Treatments often includes radiation therapy to lower risk of recurrence after a surgery. However, standard preoperative or postoperative radiation can cause significant side effects, and it is unclear which method is best. This study looked at how a well a shorter, lower-dose of preoperative radiation combined with a small dose radio-sensitizing chemotherapy compared to standard treatments. Using data from all sarcoma patients treated in our province over the span of 12 years, we found that this approach may provide similar outcomes in terms of survival and recurrence. These findings could help guide future studies on this approach and potentially offer an alternative to current treatment approaches.

## 1. Introduction

Soft tissue sarcomas (STSs) of the extremities and trunk are a rare mesenchymal group of tumors constituting 1–2% of all solid malignant neoplasms, and their rarity warrants optimal management in specialized centers, as success at achieving durable local control is key for limb salvage [1]. The standard of care for localized STS has evolved to prioritize limb-sparing surgery in conjunction with radiation therapy (RT) to achieve optimal local control while preserving limb function [2,3,4,5]. Despite this, reported local recurrence still ranges from 7% to as high as 30% [1,2,3,4,6,7].

Radiation therapy plays a critical role in local disease management; however, its optimal timing (before vs. after surgery and dosing) remains an area of ongoing investigations. Preoperative RT has been associated with reduced treatment volumes and lower total radiation dose, but it does carry a higher risk of wound complications compared to postoperative RT. Conversely, postoperative RT, while potentially decreasing acute wound healing issues, often requires higher total doses and larger treatment volumes, increasing the risk of long-term fibrosis, joint stiffness, and radiation-induced fractures [8].

In response to the limitations of both conventional approaches, a modified version of the Eilber protocol (MEP) has been adopted at our center for over two decades [2,9]. This protocol involves three doses of radiosensitizing IV doxorubicin (30 mg/day) over 3 days, followed by 10 radiation fractions of 3 Gy over 10 days (total dose 30 Gy). Advantages of this protocol are linked to the lower dose of radiotherapy administered, which include lower major wound complications as well as good quality of life and functional outcomes [10].

Although promising results have been observed in institutional series, there remains a need to validate the MEP approach in a broader, real-world population [2,9]. This study therefore sought to assess the oncologic outcomes associated with the MEP in comparison to conventional preoperative and postoperative radiation protocols using population-based data from the Alberta Cancer Registry. The aim of the current study is to assess local control of the MEP compared to either preoperative or postoperative radiation alone in patients with STS in a population-based analysis. As a secondary outcome, we aimed to compare overall survival between groups.

## 2. Methods

### 2.1. Patients/Tumor Characteristics

All patients more than 18 years of age with a pathology diagnosis of soft tissue sarcoma (STS) of the extremities and trunk within the Alberta Cancer Registry from 2004 to 2016 were identified. This registry captures all patients diagnosed and treated with solid tissue tumors within the province. Patients with STS who received either MEP, preoperative, or postoperative radiotherapy in addition to surgery were included. Exclusion criteria were patients with retroperitoneal sarcoma location, those treated with surgery alone, STS patients with metastatic disease at initial presentation, or those with STS subtypes primarily treated with systemic chemotherapy (e.g., Ewing sarcoma). Data was extracted from the registry database and chart review. Patient characteristics including age and sex were tabulated. Tumor characteristics included tumor histology, location, grade, size, and depth.

### 2.2. Treatment

The modified Eilber protocol (MEP) includes 3 days of intravenous doxorubicin (30 mg/day) and sequential radiotherapy of 3000 cGy (300 cGy per day for 10 fraction/days). Preoperative radiotherapy consisted of 50.4 Gy given in 28 fractions over 5 weeks, and postoperative radiotherapy consisted of 66 Gy given in 33 fractions delivered over 6–7 weeks. For both the MEP and preoperative radiation groups, wide local excision with margins of ≥1cm was typically performed 4–8 weeks after the completion of radiotherapy. Narrower margins were accepted in all groups if necessary for function preservation.

All patients were discussed in multidisciplinary sarcoma rounds prior to their treatments with radiology and pathology reviewed. Treatment-related details were collected including which cohort group patients were treated in as well as pathologic margin status after surgery.

These patients were followed with interval clinical examination and imaging for a total of five years at their respective cancer centers. Patients with intermediate or high-grade STS were typically followed by clinical exam, local imaging (MRI), and chest imaging (CT chest at diagnosis and chest X-ray at follow-up) q4 months for 2 years, q6 months for the third year, and annually for a total of 5 years. Those with low-grade STS were followed similarly but q6 months × 2 years and then annually until 5 years. Additional imaging was arranged per clinician discretion for possible recurrence.

### 2.3. Statistical Analysis

Patient and tumor characteristics were compared using one-way ANOVA for continuous variable and chi-square test and Fisher test for the categorical outcomes. Local recurrence-free survival and overall survival were analyzed using Kaplan–Meier curves with the log-rank test to determine differences between groups.

## 3. Results

A total of 242 patients met the inclusion criteria. Of these, 100 received the MEP, 91 underwent standard preoperative RT, and 51 were treated with postoperative RT. The mean age of the included patients was 56 (range: 19–92) with 56.2% male patients (136/242 patients) and 43.8% female patients (106/242 patients). The average number of follow-up years or years until death for participants was 4.94 years (±3.19 years) (95% CI: 4.55–5.32 years). The median follow-up years or until death for participants was found to be 4.91 years (Range: 0–12.5 years).

Most treated tumors were high-grade and deep, with 88.1% being grade 2 or 3 (193/242 patients) and 80.6% located beneath the fascia (195/242 patients). Additionally, 72.3% of tumors measured greater than 5cm in maximum dimension (175/242 patients) (Table 1).

In general, there were no important differences between the groups in terms of age (*p* = 0.166), sex (*p* = 0.263), and diagnosis (*p* = 0.057) (Table 1). There were, however, a few differences observed between groups regarding tumor size, margins, and tumor grade. Patients in the postoperative RT group more commonly presented with tumors less than 5 cm (49%, *p* < 0.001), superficial tumors (39%, *p* < 0.001), and had a higher proportion of positive surgical margins (46%, *p* < 0.001). In contrast, patients in the MEP and preop RT groups more often achieved negative (R0) margins, exceeding 90% in both groups (*p* < 0.001). The MEP also had a slightly higher proportion of low-grade tumors (18% or 10/100 patients), although the majority were still intermediate or high-grade (76% or 76/100 patients) (Table 1).

Adjuvant chemotherapy was rarely used, with only four patients across all groups receiving systemic therapy post-resection (Table 1).

Local recurrences were low across all treatment groups. The risk of local recurrence alone was 10% in the MEP group (10/100 patients), 6.6% in the preoperative RT group (6/91 patients), and 7.8% (4/51 patients) in the postoperative RT group. When patients with both local and distant recurrence were included, the overall local recurrence was 9.9% (24/242 patients). When comparing groups, there were no differences in local recurrence (*p* = 0.174), suggesting that the MEP combined with surgery can achieve local control comparable to the more traditional radiation protocols (Table 1).

**Figure 1:**
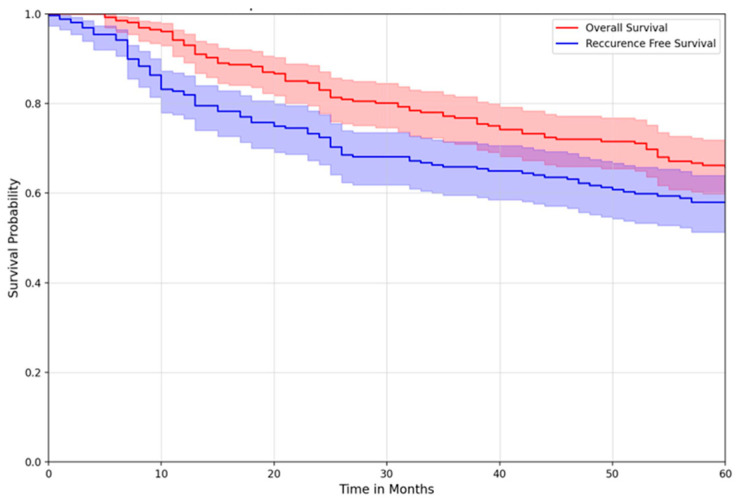
Overall survival vs. recurrence-free survival of all patients.

**Figure 2:**
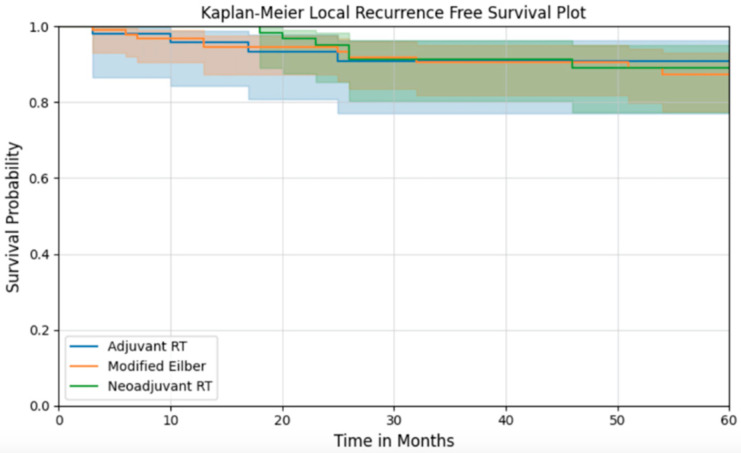
Local recurrence-free survival of patients according to their treatment group.

Overall survival was also similar across treatment groups. The five-year overall survival for the entire cohort was 65.7% (95% CI: 59.3–71.4) (Figure 1). At 5 years, recurrence-free survival for the MEP, preop RT, and postop RT groups were 87.4% (95% CI: 0.777–0.931), 89.1% (95% CI: 0.772–0.950), and 90.7% (95% CI: 0.770–0.965), respectively, with no difference between treatment groups (Figure 2).

## 4. Discussion

This population-based analysis demonstrated that the modified Eilber protocol (MEP), using a lower radiation dose over 10 days in combination low-dose doxorubicin as a radiosensitizer, was shown to have equivalent local control and overall survival rate when compared to preoperative radiation (RT) or postoperative radiation (RT) alone in patients with soft tissue sarcomas of the extremity and trunk (STS). Local recurrence risks across all treatment groups were uniformly low, ranging from 90 to 93%, without any statistically significant differences between groups. Similarly, the MEP did not affect overall survival when compared to other protocols.

One of the central goals in managing STS is to minimize the risk of local recurrence while preserving function. Historical local recurrence ranged from 15 to 30%, but modern strategies have driven this figure down to 7–15% in many series [2,5,11]. The recurrence risk of 8.3% observed in this cohort reflects excellent contemporary outcomes.

More importantly, the utilization of a lower radiation dose over a shorter course in the MEP continues to have clinical equipoise when compared to adjuvant and neoadjuvant RT. In addition, no trial thus far has shown differences in local recurrence between preoperative or postoperative radiation in soft tissue sarcoma of the extremity or trunk [8]. Potential benefits of preoperative low-dose chemoradiation may include decreased tissue toxicity, namely, fibrosis, treatment-related fractures, and major wound complication risks [2,5,10].

The use of low-dose doxorubicin as a radiosensitizer in the MEP did not preclude the later use of systemic therapy. However, adjuvant chemotherapy does remain controversial due to the current lack of evidence; it is notable that only four patients in this population-based cohort had adjuvant chemotherapy after resection [12].

In addition to its clinical effectiveness, the MEP offers practical advantages that are particularly relevant in a geographically expansive province like ours. The protocol’s short duration enables patients, particularly those from rural or out-of-province locations, to limit their time away from home. An approximate 2–3 week stay in the city can be planned, including the initial 3 days of intravenous doxorubicin with sequential outpatient 10-day course of RT, offering a distinct logistical advantage over longer outpatient radiation schedules associated with conventional preoperative or postoperative approaches [9].

Preoperative radiation, whether the MEP or preoperative RT alone, appears to be preferred by our sarcoma specialists. Additional reported benefits for deep STS being treated with preoperative RT include lower treatment volume, well oxygenated tumor requiring lower overall radiation doses, as well as potential for preoperative down-staging, which increases the ability to obtain negative histological margins [13,14,15,16,17,18].

Despite its strengths, this study has limitations. The retrospective nature and lack of randomization could have introduced potential biases. Even though data was prospectively collected from the Alberta Cancer Registry, detailed information about wound complications were missing, and thus, we were not able to report on this for the current study. In addition, some patients from outside the province may have been lost to long-term follow-up, although this represents a small minority of the cohort. Nonetheless, this study’s major strengths lie in its comprehensive, population-based approach, encompassing a full spectrum of real-world patients treated across our province.

## 5. Conclusions

The modified Eilber protocol may offer similar results to the other standard approaches in the treatment of extremity sarcomas with similar low local recurrence risk and no differences in overall survival data. Our center is likely to continue using the modified Eilber protocol given previously described benefits, which include lower radiation doses while maintaining oncologic equivalence. Given the geographic size of our province, the MEP can provide logistical benefits to our patients including less travel time and time away from home.

## Figures and Tables

**Table 1:** Patients’ characteristics, tumor characteristics, and recurrence patterns during this study period.

		Grouped by Protocol Applied					
		Missing	Overall	Adjuvant RT	Modified Eilber	Neoadjuvant RT	*p*-Value
*n*			242	51	100	91	
age at diagnosis, mean (95% CI)		0	56.0	52.9 (47.9–57.9)	55.4 (52.2–58.6)	58.4 (54.7–62.1)	0.166
sex, *n* (%)	Female	0	106 (43.8)	27 (52.9)	39 (39.0)	40 (44.0)	0.263
	Male		136 (56.2)	24 (47.1)	61 (61.0)	51 (56.0)	
final path/histology code, *n* (%)	Undifferentiated pleomorphic sarcoma	0	50 (20.7)	5 (9.8)	29 (29.0)	16 (17.6)	0.057
	Liposarcoma		37 (15.3)	8 (15.7)	14 (14.0)	15 (16.5)	
	Synovial cell sarcoma		27 (11.2)	3 (5.9)	13 (13.0)	11 (12.1)	
	Leiomyosarcoma	0	20 (8.3)	4 (7.8)	5 (5.0)	11 (12.1)	
	Other *		108 (44.6)	31 (60.8)	39 (39.0)	38 (41.8)	
tumor location code, *n* (%)	Limb girdle	0	26 (10.7)	6 (11.8)	9 (9.0)	11 (12.1)	0.044
	Lower limb distal		44 (18.2)	10 (19.6)	22 (22.0)	12 (13.2)	
	Lower limb proximal		111 (45.9)	16 (31.4)	49 (49.0)	46 (50.5)	
	Trunk		23 (9.5)	7 (13.7)	4 (4.0)	12 (13.2)	
	Upper limb distal		24 (9.9)	10 (19.6)	9 (9.0)	5 (5.5)	
	Upper limb proximal		14 (5.8)	2 (3.9)	7 (7.0)	5 (5.5)	
recurrence, *n* (%)	Distant	0	67 (27.7)	13 (25.5)	20 (20.0)	34 (37.4)	0.174
	Local		20 (8.3)	4 (7.8)	10 (10.0)	6 (6.6)	
	Local and distant		4 (1.7)		2 (2.0)	2 (2.2)	
	No recurrence		151 (62.4)	34 (66.7)	68 (68.0)	49 (53.8)	
tumor grade, *n* (%)	1	0	26 (10.7)	2 (3.9)	18 (18.0)	6 (6.6)	0.032
	2		67 (27.7)	13 (25.5)	32 (32.0)	22 (24.2)	
	3		126 (52.1)	30 (58.8)	44 (44.0)	52 (57.1)	
	9		23 (9.5)	6 (11.8)	6 (6.0)	11 (12.1)	
tumor size code, *n* (%)	0–5	1	66 (27.4)	25 (49.0)	28 (28.0)	13 (14.4)	<0.001
	5–10		102 (42.3)	17 (33.3)	47 (47.0)	38 (42.2)	
	>10		73 (30.3)	9 (17.6)	25 (25.0)	39 (43.3)	
depth code, *n* (%)	Deep	0	195 (80.6)	31 (60.8)	90 (90.0)	74 (81.3)	<0.001
	Superficial		47 (19.4)	20 (39.2)	10 (10.0)	17 (18.7)	
margins code, *n* (%)	Negative	4	200 (84.0)	27 (54.0)	90 (90.9)	83 (93.3)	<0.001
	Positive		38 (16.0)	23 (46.0)	9 (9.1)	6 (6.7)	

* for sarcoma subtypes in category Other, see Table 2.

**Table 2:** Listing of the sarcomas within the category Other.

Subtypes of Sarcoma	*n*
Spindle cell sarcoma (unknown subtype)	20
Myxofibrosarcoma	19
Fibromyxosarcoma	9
Myxoid chrondrosarcoma	7
Myxoid sarcoma	7
Dermatofibrosarcoma protuberans with sarcomatous transformation	7
Malignant peripheral nerve sheath tumor	5
Epitheloid sarcoma	4
Ewing sarcoma	4
Epithelioid hemangioendothelioma	3
Myxoinflammatory fibroblastic sarcoma	3
Clear cell sarcoma	3
Extraskeletal myxoid chondrosarcoma	3
Angiosarcoma	2
Myofibroblastic tumor	2
Pleomorphic sarcoma	1
Rhabdomyosarcoma	1
Undifferentiated round cell sarcoma	1
Epitheliod fibrosarcoma	1
Extraskeletal osteosarcoma	1
Giant cell sarcoma	1
Mesenchymal chodrosarcoma	1
Embryonal spindle cell rhabdomyosarcoma	1
Rhabdoid tumor	1
Malignant myoepithelioma	1

## Data Availability

The datasets presented in this article are not readily available due to institutional and organizational data-sharing restrictions within the province related to patient privacy. Requests to access the datasets should be directed to the corresponding author.
